# Supplementary material for: Green Assessment of Phenolic Acid Composition and Antioxidant Capacity of Advanced Potato Mutant Lines through UPLC-qTOF-MS/MS Quantification
Source: Foods. 2023 Jul 6;12(13):2616. doi: 10.3390/foods12132616 (PMC10340226; doi:10.3390/foods12132616)
Supplement: Supplementary file 1 [file foods-12-02616-s001.zip › foods-2464734-supplementary.pdf]

Supplementary

Green Assessment of Phenolic Acid Composition and Antioxidant Capacity of Advanced Potato Mutant Lines through UPLC-qTOF-MS/MS Quantification

Sub-Title: Evaluation of potato mutant (M_1V_8) lines compared to the corresponding parents by quantification of identified phenolics and antioxidant capacity in tubers

Clara Gomez-Urios [1], Hristo Kalaydzhiev [2], Jesus Blesa [1], Maria Jose Esteve [1], Emiliya Nacheva [3], Dida Iserliyska [4] and Nasya Tomlekova [3,*]

[1] Nutrition and Food Science, Faculty of Pharmacy, University of Valencia, Avenida Vicent Andrés Estellés, s/n, 46100 Burjassot, Spain; clara.urios@uv.es (C.G.-U.); jesus.blesa@uv.es (J.B.); maria.jose.esteve@uv.es (M.J.E.)
[2] Department of Analytical Chemistry and Physical Chemistry, University of Food Technologies—Plovdiv, 26 Maritsa Blvd., 4002 Plovdiv, Bulgaria; hristo.kalaydzhiev@yahoo.com
[3] Maritsa Vegetable Crops Research Institute, Agricultural Academy—Sofia, 32 Brezovsko shosse St, 4003 Plovdiv, Bulgaria; emnach@abv.bg
[4] Institute of Food Preservation and Quality—Plovdiv, Agricultural Academy—Sofia, 154 Vasil Aprilov Blvd, 4003 Plovdiv, Bulgaria; dida_isser@yahoo.com
* Correspondence: nasia.tomlekova@gmail.com; Tel.: +359-(0)32-951227

Table S1: Quantity of phenolic compounds in tubers of parents, controls and advanced potato mutant lines (M_1V_8) – summarized view.

			Phenolic acid, (µg/mL)											
			**FA**	**RT**	**NIN**	**API**	**HET**	**CGA**	**QUE**	**NR**	**HSP**	**CA**	**NAR**	**KMP**
**Quantity of particular phenolic compound in samples, µg/mL**	**Parent plants**	**PC 428**	0.1145 ± 0.0004	N/D	N/D	0.0430 ± 0.0004	N/D	12.5750 ± 0.0028	N/D	N/D	0.0275 ± 0.0004	5.1700 ± 0.0020	0.0075 ± 0.0003	0.0015 ± 0.0001
		**PC 490**	0.0205 ± 0.0003	N/D	N/D	0.0445 ± 0.0009	N/D	3.7970 ± 0.0009	N/D	N/D	0.0710 ± 0.0010	N/D	0.0070 ± 0.0002	0.0015 ± 0.0001
		**PC 538**	N/D	N/D	N/D	0.0410 ± 0.0007	N/D	2.4010 ± 0.0011	N/D	N/D	N/D	N/D	0.0070 ± 0.0004	0.0015 ± 0.0002
		**PC 707**	0.0230 ± 0.0004	0.0110 ± 0.0001	N/D	0.0440 ± 0.0002	N/D	3.3095 ± 0.0012	N/D	N/D	0.0120 ± 0.0003	0.5425 ± 0.0009	0.0035 ± 0.0001	0.0015 ± 0.0001
		**PC 757**	0.0480 ± 0.0006	N/D	N/D	0.0440 ± 0.0004	N/D	1.8965 ± 0.0005	N/D	N/D	0.0635 ± 0.0010	N/D	0.0065 ± 0.0003	0.0010 ± 0.0001
	**Controls**	**K-III-2**	0.0565 ± 0.0004	0.2020 ± 0.0008	0.0040 ± 0.0003	0.0465 ± 0.0007	0.0260 ± 0.0004	6.8700 ± 0.0007	N/D	N/D	0.1025 ± 0.0010	N/D	0.0035 ± 0.0002	0.0130 ± 0.0006
		**K-IV-3**	0.0290 ± 0.0007	N/D	N/D	0.0595 ± 0.0004	N/D	2.1180 ± 0.0008	0.0470 ± 0.0008	N/D	0.0025 ± 0.0003	N/D	0.0065 ± 0.0003	0.0010 ± 0.0001
		**K-VII-4**	0.0310 ± 0.0002	0.0060 ± 0.0002	N/D	0.0405 ± 0.0003	N/D	2.7125 ± 0.0012	N/D	N/D	0.0045 ± 0.0001	N/D	N/D	0.0030 ± 0.0001
	**Mutants**	**M-I-8**	0.0210 ± 0.0002	0.0075 ± 0.0005	N/D	0.0985 ± 0.0005	N/D	2.2790 ± 0.0010	0.1015 ± 0.0005	0.0080 ± 0.0004	0.0325 ± 0.0003	N/D	0.0080 ± 0.0002	0.0025 ± 0.0002
		**M-I-17**	N/D	0.0240 ± 0.0004	N/D	0.0430 ± 0.0003	0.0180 ± 0.0005	1.7350 ± 0.0006	N/D	0.0085 ± 0.0003	0.0590 ± 0.0006	N/D	0.0080 ± 0.0002	0.0010 ± 0.0002
		**M-III-8**	0.0220 ± 0.0005	0.0295 ± 0.0001	N/D	0.0400 ± 0.0006	N/D	1.9030 ± 0.0006	0.0170 ± 0.0002	0.0160 ± 0.0007	0.1100 ± 0.0013	0.0700 ± 0.0005	0.0045 ± 0.0003	0.0010 ± 0.0002
		**M-III-9**	0.0655 ± 0.0005	0.0025 ± 0.0003	N/D	0.0395 ± 0.0004	N/D	1.6415 ± 0.0014	N/D	N/D	0.0200 ± 0.0002	N/D	0.0060 ± 0.0003	0.0015 ± 0.0002
		**M-III-30**	0.0800 ± 0.0005	0.0795 ± 0.0007	N/D	0.3200 ± 0.0013	N/D	2.8435 ± 0.0008	0.4165 ± 0.0010	N/D	0.0580 ± 0.0005	0.5090 ± 0.0014	0.0180 ± 0.0004	0.0080 ± 0.0002
		**M-III-48**	0.0895 ± 0.0005	0.0110 ± 0.0004	0.0015 ± 0.0002	0.0455 ± 0.0004	0.0155 ± 0.0007	7.5400 ± 0.0021	0.0170 ± 0.0004	0.0405 ± 0.0004	0.1025 ± 0.0007	N/D	0.0210 ± 0.0002	0.0010 ± 0.0003
		**M-III-50**	0.0560 ± 0.0005	0.0150 ± 0.0005	N/D	0.0430 ± 0.0004	N/D	9.2200 ± 0.0021	0.0185 ± 0.0003	N/D	0.0350 ± 0.0002	1.4905 ± 0.0007	0.0040 ± 0.0001	0.0015 ± 0.0002
		**M-IV-14**	0.0095 ± 0.0004	0.0010 ± 0.0003	N/D	0.0455 ± 0.0007	N/D	0.7860 ± 0.0008	0.0305 ± 0.0004	N/D	N/D	0.1185 ± 0.0006	0.0030 ± 0.0002	0.0025 ± 0.0002
		**M-IV-17**	0.0220 ± 0.0003	0.0040 ± 0.0005	N/D	0.0410 ± 0.0004	N/D	2.0435 ± 0.0006	0.0200 ± 0.0002	N/D	N/D	N/D	0.0050 ± 0.0003	0.0015 ± 0.0002
		**M-VII-7**	0.0585 ± 0.0001	0.0035 ± 0.0003	N/D	0.0385 ± 0.0005	N/D	1.9860 ± 0.0009	N/D	N/D	0.0175 ± 0.0003	0.0140 ± 0.0004	0.0035 ± 0.0002	0.0015 ± 0.0001

Abbreviations: FA – ferulic acid, RT – rutin, NIN – naringin, API – apigenin, HET – hesperetin, CGA – chlorogenic acid, QUE – quercetin, NR – narirutin, HSP – hesperidin, CA – trans-cinnamic acid, NAR – naringenin, and KMP – kaempferol. N/D – Not detected.

Table S2. Data for antioxidant activity received by homogeneous subsets from One-way ANOVA with Tukey's test for all samples studied.

Method/Test	Sample	N	Subset for alpha = 0.05						
			1	2	3	4	5	6	7
Tukey HSD[a]	PC 428	3	1.4497192						
	M-III-30	3	1.5466196	1.5466196					
	PC 707	3	1.8361749	1.8361749	1.8361749				
	PC 538	3	1.9649305	1.9649305	1.9649305	1.9649305			
	M-IV-14	3	2.0221159	2.0221159	2.0221159	2.0221159			
	K-III-2	3	2.0705589	2.0705589	2.0705589	2.0705589			
	PC 490	3	2.0870526	2.0870526	2.0870526	2.0870526			
	K-IV-3	3	2.1649354	2.1649354	2.1649354	2.1649354	2.1649354		
	PC 757	3	2.4160750	2.4160750	2.4160750	2.4160750	2.4160750	2.4160750	
	M-I-17	3	2.6110475	2.6110475	2.6110475	2.6110475	2.6110475	2.6110475	
	M-III-8	3		2.7323233	2.7323233	2.7323233	2.7323233	2.7323233	2.7323233
	K-VII-4	3			2.7920872	2.7920872	2.7920872	2.7920872	2.7920872
	M-IV-17	3				3.0324648	3.0324648	3.0324648	3.0324648
	M-VII-7	3				3.0791689	3.0791689	3.0791689	3.0791689
	M-I-8	3					3.3184088	3.3184088	3.3184088
	M-III-50	3						3.4108807	3.4108807
	M-III-9	3							3.8091633
	M-III-48	3							3.8425851
	Sig.		.061	.050	.243	.086	.064	.192	.088

Table S3. Validation parameters of UPLC-qTOF-MS/MS method.

№	Analytes	Molecular formula	*m/z*	[*m*-H]	Error (ppm)	R^2
1	Quercetin	$C_{15}H_{10}O_7$	302,04265	301,03538	0,4	0.9997
2	Chlorogenic acid	$C_{16}H_{18}O_9$	354,09508	353,08781	-0,7	0.9942
3	Rutin	$C_{27}H_{30}O_{16}$	610,15339	609,14611	-0,9	0.9999
4	Ferulic acid	$C_{10}H_{10}O_4$	194,05791	193,05063	-0,2	0.9992
5	Narirutin	$C_{27}H_{32}O_{14}$	580,17921	579,17193	-1,2	0.9986
6	Naringin	$C_{27}H_{32}O_{14}$	580,17921	579,17193	-1,2	0.9986
7	Hesperidin	$C_{28}H_{34}O_{15}$	610,18977	609,18249	-0,9	0.9999
8	*trans*-cinnamic acid	$C_9H_8O_2$	148,05243	147,04515	0,6	0.9991
9	Naringenin	$C_{15}H_{12}O_5$	272,06847	271,0612	0,7	0.9998
10	Apigenin	$C_{15}H_{10}O_5$	270,05282	269,04555	1,1	0.9986
11	Hesperetin	$C_{16}H_{14}O_6$	302,07904	301,07176	-0,9	0.9996
12	Kaempferol	$C_{15}H_{10}O_6$	286,04774	285,04046	1,4	0.997
